# A Statistical Framework for the Interpretation of mtDNA Mixtures: Forensic and Medical Applications

**DOI:** 10.1371/journal.pone.0026723

**Published:** 2011-10-28

**Authors:** Thore Egeland, Antonio Salas

**Affiliations:** 1 Department of Chemistry, Biotechnology and Food Science, Norwegian University of Life Sciences, Oslo, Norway; 2 Unidade de Xenética, Instituto de Medicina Legal and Departamento de Anatomía Patolóxica e Ciencias Forenses, Facultade de Medicina, Universidade de Santiago de Compostela, Galicia, Spain; Charité Universitätsmedizin Berlin, NeuroCure Clinical Research Center, Germany

## Abstract

**Background:**

Mitochondrial DNA (mtDNA) variation is commonly analyzed in a wide range of different biomedical applications. Cases where more than one individual contribute to a stain genotyped from some biological material give rise to a mixture. Most forensic mixture cases are analyzed using autosomal markers. In rape cases, Y-chromosome markers typically add useful information. However, there are important cases where autosomal and Y-chromosome markers fail to provide useful profiles. In some instances, usually involving small amounts or degraded DNA, mtDNA may be the only useful genetic evidence available. Mitochondrial DNA mixtures also arise in studies dealing with the role of mtDNA variation in tumorigenesis. Such mixtures may be generated by the tumor, but they could also originate *in vitro* due to inadvertent contamination or a sample mix-up.

**Methods/Principal Findings:**

We present the statistical methods needed for mixture interpretation and emphasize the modifications required for the more well-known methods based on conventional markers to generalize to mtDNA mixtures. Two scenarios are considered. Firstly, only categorical mtDNA data is assumed available, that is, the variants contributing to the mixture. Secondly, quantitative data (peak heights or areas) on the allelic variants are also accessible. In cases where quantitative information is available in addition to allele designation, it is possible to extract more precise information by using regression models. More precisely, using quantitative information may lead to a unique solution in cases where the qualitative approach points to several possibilities. Importantly, these methods also apply to clinical cases where contamination is a potential alternative explanation for the data.

**Conclusions/Significance:**

We argue that clinical and forensic scientists should give greater consideration to mtDNA for mixture interpretation. The results and examples show that the analysis of mtDNA mixtures contributes substantially to forensic casework and may also clarify erroneous claims made in clinical genetics regarding tumorigenesis.

## Introduction

There are a number of different areas where mitochondrial DNA (mtDNA) is of great relevance, including molecular anthropology, population genetics, and clinical and forensic genetics. Here we deal with the topic of mtDNA mixtures and we show a statistical framework for mixture interpretation that is of particular interest in forensic genetics and medical genetic studies. The analysis of mixtures in forensic casework traditionally relies on the genotyping of a set of autosomal Short Tandem Repeats (STRs) that are generally well standardized in commercial kits. Y-chromosome markers are particularly useful in rape cases because they specifically target the DNA contribution from the male aggressor without interference from the female victim. However, the current procedures have several limitations. For instance, it is often difficult to determine the number of contributors in cases involving degraded samples with many contributors, or when the individuals contribute similar amounts to the mixture. Mitochondrial DNA is particularly well suited for the analysis of degraded samples, especially due to the high proportion of mtDNA molecules compared to the nuclear DNA. Mitochondrial DNA can be analyzed in cases where other DNA sources fail; for instance, in hair shafts or in samples containing low amounts of DNA.

In oncogenetic studies relevant claims about the implications of mtDNA somatic mutations (instabilities) in tumorigenesis were several times formulated [1], [2], [3]. Before such claims are made, reasonable alternative explanations should be ruled out. For instance, the data could result from an inadvertent mixture, implying that the tumor sample was contaminated by exogenous DNA from some other individual [4], [5]. Combining phylogenetic knowledge with proper statistical analyses, as discussed in this paper, it is possible to unravel the origin and nature of biological contamination of PCR amplicons and involuntary sample mix-ups. These unfortunate artifacts have undermined a field of research dealing with the analysis of mtDNA instability in cancer [4], [5]. The evidentiary samples commonly analyzed in the forensic field are often degraded or contain low amounts of DNA; therefore, contamination has been one of the most important ‘hobby horses’ in forensic casework.

On the other hand, current autosomal STR assays have limitations when attempting to determine the most likely origin of a profile; the role of SNPs is more promising [6], but little has been done to date in order to replace STRs with SNPs in this field of research [7], [8]. Mitochondrial DNA is strongly stratified in human populations, therefore it is possible to determine the most likely geographical origin of an mtDNA profile [9], at least on a continental scale, although the level of geographical resolution depends, for instance, on the mtDNA markers targeted. Contributors to a mixture may come from different populations and therefore the analysis of mtDNA could be useful in orienting police investigations. The main limitation here comes from the fact that, in reality, mtDNA is a single marker and does not fully represent the complete genome of an individual.

To the best of our knowledge, there are only three relevant studies in our context that have empirically evaluated the ability of mtDNA to resolve mixtures. Walker et al. [10] used mtDNA variations to estimate the number of contributors to a mixture that was artificially created by combining different mtDNA profiles. The study by Montesino et al. [11] was a collaborative multi-centric exercise organized by the GEP-ISFG (Spanish and Portuguese Group of the International Society for Forensic Genetics) aimed at analyzing mtDNA sequence patterns in different sorts of mixed stains from different biological sources, namely saliva, semen, and blood. The empirical results obtained from several laboratories pointed to the potential of mtDNA to disentangle mixtures. However, in the two studies mentioned [11] there was no attempt to create a statistical framework. More recently, Holland et al. [12] have used second generation sequencing for mtDNA mixture deconvolution and for the detection to a high resolution of mtDNA heteroplasmies. The results of the analysis in Holland's et al. indicate that “*the ability to routinely deconvolute mtDNA mixtures down to a level of 1∶250 allows for high resolution analysis fo mtDNA heteroplasmy, and for differentiation of individuals from the same maternal lineage*”. In addition, analyses of mtDNA mixtures due to contamination in single cell analysis have been also carried out in Yao et al. [13].

We have previously highlighted the relevance of using phylogenetic characteristics of the mtDNA molecule in other forensic applications. The specific inheritance features of the non-recombining mtDNA molecule allow a natural grouping of sequence haplotypes into principal monophyletic clades, referred to as haplogroups [14]. The interplay between phylogeny and forensic genetics [15] could be also of interest when applied to the deconvolution of mtDNA mixtures. The focus of the present paper is on the methods, but we will illustrate using simulated and real data. The methods depend heavily on the amount of information available in the trace, ranging from full quantitative information to categorized coding (see below). The appropriate methods, depending on the data available, are discussed and some preliminary implementations are presented. There are three important problems that we will address in turn: (i) the deconvolution of mixtures; (ii) the weighting of evidence, and (iii) a general assessment of the informative value in mtDNA mixtures. These problems correspond to similar ones that have already been discussed for autosomal STRs and we will emphasize the specific features related to mtDNA mixtures. We show that important results beyond the reach of autosomal markers and Y-chromosome data can be obtained.

## Methods

### Nature of the data

The data can be split into several parts: the specific trace or stain, potential reference samples and databases. In addition, the context of the case is important, but this will not be discussed here; see [16] for a general discussion. The first part of the data, the trace, can, in principle, be available containing varying degrees of information, namely, categorized and/or quantitative data. Categorized data refers to an ambiguous sequence status where more than one nucleotide variant is observed at the same position. This positional status of the nucleotide is referred by an IUPAC international code where unique letters are given to a combination of two or more possible nucleotides (e.g. “R” means the coexistence of an adenine A and a guanine G at the same nucleotide position, see http://www.dna.affrc.go.jp/misc/MPsrch/InfoIUPAC.html). Quantitative data refers to a sequence status where the contribution of different nucleotide variants to the same position can be quantitatively measured (see the example in Table 1). Quantitative information is not generally available for the software currently used in automatic sequencers, unlike the case for other markers including STRs and SNPs. However, we believe that such data could also be provided for mtDNA and it would then be easy to perform controlled experiments to assess the validity and reliability of such data. By providing methods, exemplified using simulated data, we hope to encourage suppliers to also provide quantitative data. Our ambition has not been to go into the technical issues of quantification, but we realize that there is a need for further studies.

**Table 1:** Excerpts of the HVS-I mtDNA data; the sequence range is from position 16024 to position 16365.

Region	Sample ID	Profile (HVS-I)
Iberia	20	rCRS
Iberia	21	16093
Iberia	22	16093 16189 16293
Iberia	23	16093 16224 16311
…		
Iberia	1263	16093 16293
Iberia	1264	16069 16126
…		
Iberia	2135	16093 16189 16224 16311
Iberia	2136	16093 16224 16311
…		
Iberia	2575	16126 16294 16296 16304

Nomenclature of mtDNA variants is according to Andrews et al. [31]; the numbers indicate transitions with respect to Andrews et al. [31].

Heteroplasmies are possible in mtDNA sequences and this DNA status could lead to interpretational problems (see below). From a practical point of view, it is not possible to differentiate heteroplasmies from mixture patterns. However, heteroplasmic positions correspond to mutational events (more than one heteroplasmic position in a control region profile is uncommon) and quite often coincide with those positions that have a high mutation rate [17], and this information could be implemented in a Bayesian framework.

Table 2 shows Table 1 in a different format with quantitative information simulated and added in the two rightmost columns. For this example, a mixture was formed by haplotypes H22 and H23. The column y0 records the signal strength and peak height (or area) corresponding to the 0 allele. The corresponding figure for the 1 allele is y1 and the measurements are scaled so that y0+y1 = 1. The haplotype H22 contributes 30% and the remaining 70% comes from H23. For instance, the value for site 16224, y0 = 0.32, deviates slightly from the theoretic value of 0.3 because of some noise in the data.

**Table 2:** Table 1 with quantitative information.

Polymorphism	H20	H21	H22	H23	H1263	H1264	H2135	H2136	H2575	y1
rCRS	1	0	0	0	0	0	0	0	0	0.00
16069	0	0	0	0	0	1	0	0	0	0.00
16093	0	1	1	1	1	0	1	1	0	1.00
16126	0	0	0	0	0	1	0	0	1	0.00
16189	0	0	1	0	0	0	1	0	0	0.30
16224	0	0	0	1	0	0	1	1	0	0.68
16293	0	0	1	0	1	0	0	0	0	0.29
16294	0	0	0	0	0	0	0	0	1	0.02
16296	0	0	0	0	0	0	0	0	1	0.00
16304	0	0	0	0	0	0	0	0	1	0.00
16311	0	0	0	1	0	0	1	1	0	0.73

The polymorphisms are the transitions referred to in Andrews et al. [31]. The column 1 denotes the peak height corresponding to allele 1.

### Deconvoluting the mixture

The starting point is a stain and an mtDNA profile. The challenge of deconvolution is determining which haplotypes may have contributed to forming the stain. Note that in general, not all combinations of variants are equally possible because most of them might not make sense in a phylogenetic context. A deep knowledge of the phylogeny is therefore mandatory. We assume that all possible haplotypes are included in a database or a candidate set. Without this reasonable assumption, any stain could be explained by a hitherto unobserved haplotype. In some cases, there will be both known and unknown contributors. The most usual case involves a known, typed, victim and an unknown, un-typed, perpetrator. The general framework does not principally depend on whether or not there are some known contributors.

For categorized data, there is generally not much to say of the methods: for a given stain and a given number of contributors, we can determine the combinations of haplotypes consistent with the stain. However, computational issues could remain in the sense that the search for possible combinations can be optimized. Such computational issues are not addressed here. The result of a search may have three outcomes:

1. No solution is found.
2. Just one solution emerges.
3. Several combinations are possible.

There may be several reasons for alternative 1 above, including problems relating to contamination and the database not being exhaustive. There are different reference databases available for forensic and clinical geneticist. For instance, there are more than 150,000 partial control region segments available in the literature, some populations groups being much better represented than others (e.g. Europe is particularly well sampled). There are some other important databases that are freely accessible to the public (e.g. EMPOP: http://empop.org/). In addition, most of the forensic laboratories have their in-house databases for internal use in their own forensic casework.

Alternative 2 is the simplest, where only the strength of the evidence remains to be assessed, as discussed below.

Alternative 3 may sometimes be resolved if the number of contributors is known. For instance, if there is external evidence to prove that there can only be two contributors and there is only one solution corresponding to two contributors, the problem of ambiguity goes away. If, however, there are several solutions, it is impossible to distinguish between these unless further data is available. Next, we discuss how this can be done provided quantitative information is available.

A different statistical approach is then needed. To highlight the problem, consider the data of Table 1 and assume a mixture is formed by haplotypes H22 and H23. This mixture cannot be distinguished from a mixture based on H1263 and H2135 if only qualitative information is used. If, however, quantitative information is available, the problem can be solved. Table 2 shows simulated data with quantitative information corresponding to a mixture where 22 contributes a fraction of β = 0.3 and 22 a fraction of 1- β = 0.7. The column y1 gives the peak height (or area) corresponding to the 1 allele. The peak heights are scaled so they add up to 1. We first assume there is no drop out or drop in and present the general method for solving this based on the intuitively reasonable regression model:
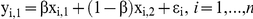
where *i* refers to the marker and 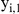 is the peak height for allele 1. Moreover, 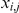 is the number of 1alleles of the contributor j ( = 1 or 2) and 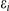 is the error term.The error term can be pragmatically be modeled as a normal distribution truncated to non-negative values. Observe that we model only the peak height for allele 1 as we assume the peak heights of the two alleles are scaled so that there is no additional information in the peak height of the other allele. More sophisticated models may be developed once more real data becomes available. The model can be rewritten as:
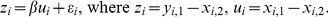
(1)


Based on this model, several problems can be solved. First observe that the hypothesis:

- H_0_: “Individual 1 did not contribute to the mixture”

is equivalent to testing H_0_: 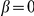. This can be done using a statistical package. In this case standard linear regression formulae are also available. For instance, the least square estimate of the fraction is
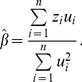
(2)Furthermore, the competing hypotheses can be distinguished based on how well the data fits. Specifically, we can compute the model fit as measured by the conventional 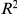 and the most likely hypothesis is the one with the largest 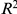. For the example in this section, 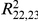 corresponds to the model fit assuming individuals with haplotypes 22 and 23 contributed. The corresponding figure if the mixture was formed by individuals with haplotypes 1263 and 2135 is 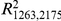 and the most likely explanation corresponds to the alternative with the larger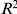. The calculations can be performed using standard statistical software, such as R, or standard formulae.

Next, we extend the model (1) to account for drop in and drop out. Let:
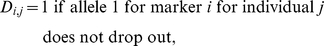
(3)and 0 otherwise. Also,
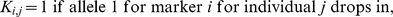
and 0 otherwise. Then, the extended version of Eq. (1) becomes
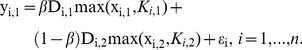
(4)An interesting question now is: if data are simulated from model (4) with realistic probabilities of drop out and drop in, will the model we use for estimation (1) still provide reasonable answers and solve the problem? Example 2 in the Results section deals with this. The assumptions of the model and more general alternatives are described in the Discussion section.

### Weighing the evidence

In principle, evidence based on mtDNA profiles is evaluated in the same way as other autosomal DNA. At least two hypotheses must be formulated. In crime cases, the prosecution's hypothesis is denoted *H_P_* and a typical version is “the mixture comes from a *lineage* corresponding to the victim and a *lineage* corresponding to the suspect”. It is essential to note that *lineage* is emphasized to indicate that mtDNA cannot be linked to individuals as all persons belonging to the same lineage will have the same mtDNA profile. The defense hypothesis is typically *H_D_*: “the mixture comes from a *lineage* corresponding to the victim and a *lineage* corresponding to the suspect”. In clinical cases and other non-court cases it is not reasonable to refer to prosecution and defense and the hypotheses are denoted *H_1_* and *H_2_ instead*. The evidence is measured by the likelihood ratio 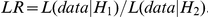 There may be more than two hypotheses, in which case several *LR* values can be calculated depending on the context. The estimates of 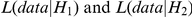 depend on the choice of database and are not discussed in any detail in this paper.

### Probability of an informative mixture

Mixtures of identical mtDNA profiles will not be informative, i.e., a mixture is unidentifiable. It is of interest to estimate the probability that a specific case will not lead to an informative mtDNA mixture. This probability will obviously depend on the database. Assuming that there are different *k* profiles with frequencies *p_1_,….,p_k_*, the probability that a mixture of a random sample of be *m>1 profiles* will be informative in the sense that not all are identical is:
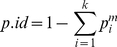
(6)This probability can also be estimated from simulations. Note that this probability strongly depends on the population group represented by the database and the range of sequence information targeted. For instance, sub-Saharan African lineages are generally more divergent and therefore more informative than European ones, and control region data may show little resolution in, for example, some Native American populations.. The calculations can be performed using the R-library unseen2 library freely available from http://folk.uio.no/thoree/nhap/.

## Results

### Example 1: on categorized data

Consider Table 1. Assume a stain is observed with transitions at sites 16189 and 16293, displaying a mixture. The only two person mixture consistent with this finding consists of haplotypes H21 and H22. We first formulate the hypotheses:

H_P_: *The mix is formed by two individuals, one from the H21 haplotype and one from the H22 haplotype,*
H_D_: *The mix is formed by two random individuals.*

Obviously, P(data| H_P_) = 1. Based on Table 1, we estimate the probability of both haplotypes as 1/11 and therefore:
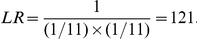
Similar calculations are performed if the hypotheses are specified differently, for instance if the individual with haplotype H21 is a known contributor, *LR = 11*. Also, in principal, nothing will change if the deconvolution and the haplotype estimates are based on larger, more realistic databases. For instance, if haplotype H21 is observed 12 times in a database of 2575 haplotypes and haplotype H22 is only observed once, this will lead to:
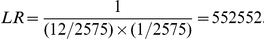
The above example provides a unique combination. The problem of deconvolution and following *LR* calculations becomes more complex if there are several solutions and several combinations consistent with the mixture. Such ambiguous situations can arise if there are possibly more than two contributors. Any haplotype with alleles coinciding with those of haplotypes H21 and H22 outside the mixture sites can be added without changing the data from the mixture.

Ambiguous problems can also arise if other mixtures are considered. For example, a mixture of H22 and H23 cannot be distinguished from a mixture of haplotypes H1263 and H2135 based on the limited number of sites shown in Table 1. Additional data or information is then needed to distinguish between the possible solutions to the deconvolution problem. The next example shows how this problem of ambiguity can be approached provided quantitative data is available.

### Example 2 (Example 1 continued): on quantitative data

Consider once again the data in Tables 1 and 2 and the hypotheses:

- *H_1_: A mixture from haplotypes H22 and H23.*
- *H_2_: A mixture from haplotypes H1263 and H2135.*

**Figure 1:**
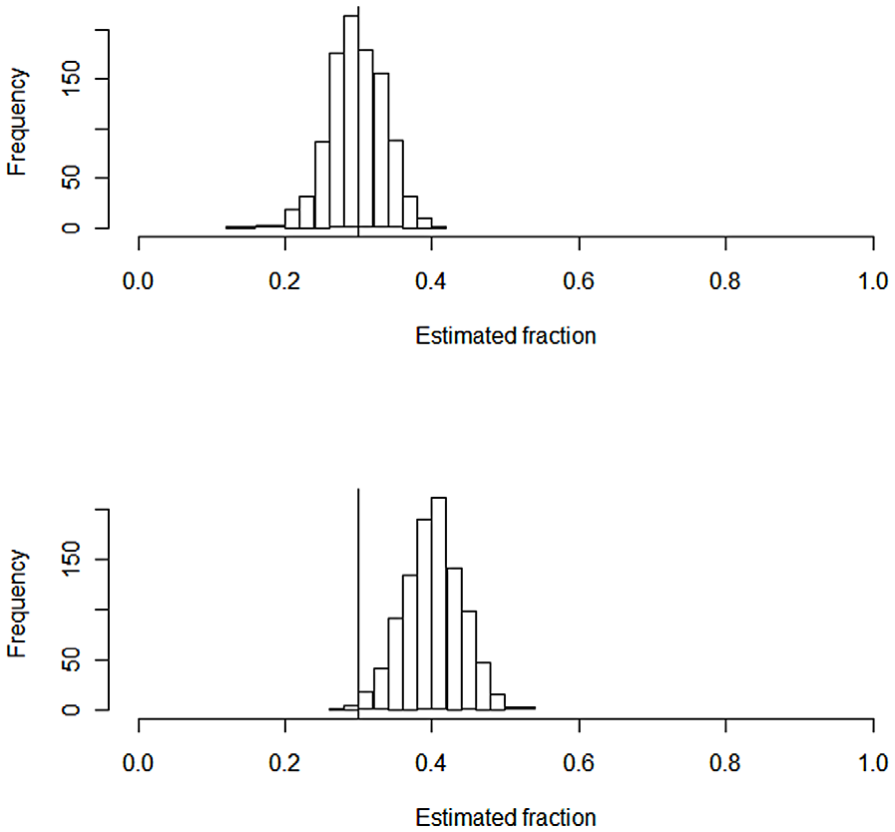
The upper panel shows that the proportion from contributor 1 is a reasonable estimate based on the correct model, whereas a clear bias appears for the wrong model. A total of 1000 simulations were performed.

**Figure 2:**
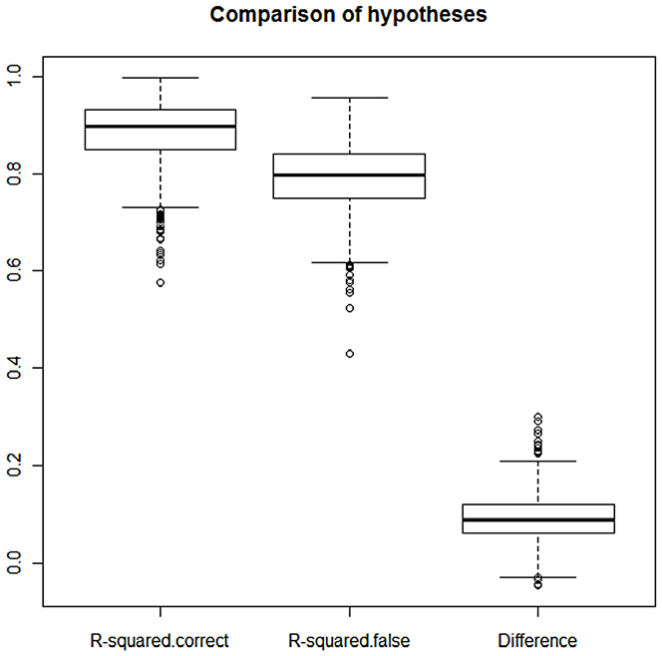
The correct model can be identified as higher values of R^2^ were obtained, as shown in the box plot on the left hand side. A pairwise comparison of the 1000 simulations performed is shown in the far right box plot.

**Figure 3:**
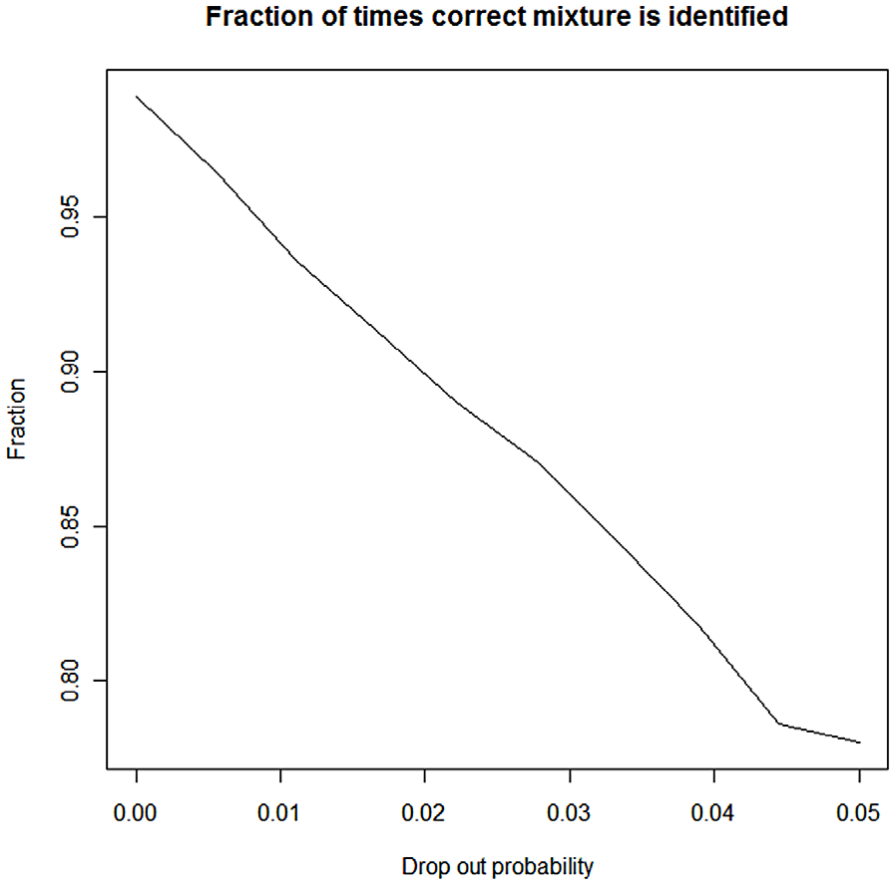
The fraction of times the correct model is identified becomes lower as the drop out probability increases, but it stays above 80% as long as the drop out probability is below 0.05.

These hypotheses are, as mentioned, indistinguishable when based on qualitative data only. To see how well we (i) *can estimate the fractions contributed* and (ii) *distinguish between the hypotheses* based on regression model (1), we simulated the peak heights. One example is displayed in the far right column of Table 2. We performed 1000 simulations. Figure 1 shows the estimated fraction from contributor 1. For the correct model the values are consistent with the true value of 0.3 with 95% of the simulated values lying between 0.22 and 0.37. Figure 2 displays the *R^2^* values for the correct model *H_1_* and the false model *H_2_* and the difference. In 98.9% of the simulations this approach concluded with the correct model. This is a promising result in view of the small data set. Figure 3 shows how the fraction of correctly identified mixtures varies as a function of the drop out probability. The data was simulated according to the model in Eq. (4) but estimated according to Eq. (1) as we assumed no knowledge of the drop out probability and thus ignored drop out in the estimation.

### Example 3: a real murder case

A real casework example is discussed below. A victim was murdered and there is a suspect and cigarette butts, which could have been shared by the victim and the suspect, according to the crime scenario. The judge presiding in the case had directly requested that the contribution by both people to the cigarette butts be revealed, as this information could be crucial for resolving the case. Analyses of autosomal STRs and SNPs failed when attempts were made to analyze the butts, and so mtDNA is the only choice for resolving the case. The profiles obtained for the HVS-I segment were:

- Victim: T16304C
- Suspect: T16126C C16292T C16294T A16399G
- Cigarette butt (mixture): 16126Y 16292Y 16294Y 16304Y 16399R

where Y in the IUPAC code refers to T/C and R to A/G.

**Figure 4:**
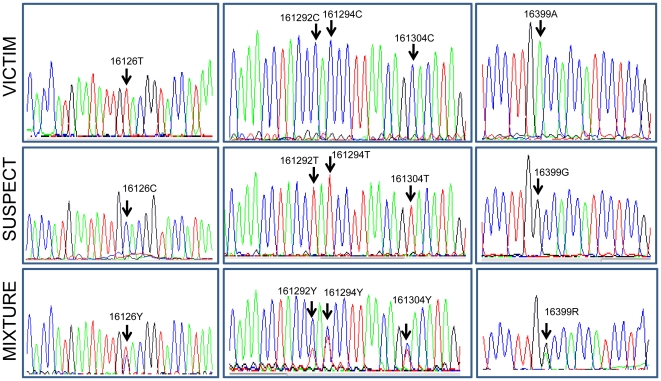
Sequencing electropherograms of the profiles discussed in example 3.

Figure 4 shows the electropherograms of the three sequencing profiles. From a phylogenetic point of view, the profile of the victim could be compatible with haplogroup H (further analysis of mtDNA SNPs [data not shown] allocated this profile to haplogroup H5). The profile of the suspect undoubtedly belongs to haplogroup T (most likely to the sub-clade T2). The mtDNA analysis of the cigarette butt shows a perfect mixture that is compatible with the presence of at least two contributors, exactly mirroring the simultaneous presence of the same profiles carried by the victim and the suspect.

The statistical evaluation starts by formulating the hypotheses:

- *H_P_: The mixture comes from the haplotypes of the victim and the suspect.*
- *H_D_: The mixture comes from the haplotypes of the victim and an unknown donor.*

In this case, the *LR = 1/p* where *p* is an estimate of the frequency of the suspect haplotype. This haplotype is not seen in a database of 2575 profiles and a conventional approach, considered favorable to the defendant, is to add the suspect's haplotype to the databases, leading to an LR of 2576.

### Example 4: in a clinical context

The procedure developed in the present study could also be used in clinical cases where mixtures of different mtDNA haplotypes frequently occur, for instance in mtDNA instability studies in cancer, where generally there is an interest in comparing a tumor sample from an affected patient with a non-tumor sample from the same patient. Discrepancies between the mtDNA profiles of these samples are generally interpreted as molecular instabilities responsible for the tumorgenesis process [1], [2]. Alternatively, such mixtures could also be consistent with artificial mixtures created accidentally by sample mix-up or contamination. This alternative hypothesis has been formulated few years ago [4], [5] and rests on solid theoretical foundations; furthermore, it has received support in other fields of genetic research, such as human population genetics [18], [19], [20], forensic genetics [15], [21], [22], [23] and other disease studies [24], [25], [26], [27]. Basically, this alternative hypothesis considers the confrontation of the instabilities observed in oncogenetic studies against the known mtDNA phylogeny (and considering positional mutational patterns [28]); most of the times, the seeming instability patterns are perfectly compatible with a contamination or sample mix-up events occurring during any step of the genotyping process (e.g. mixture of two known mtDNA haplotypes). According to the supporters of this explanation, these patterns of instabilities are difficult to reconcile with the intervention of some kind of molecular process (occurring during carcinogenesis) that is able to exactly reproduce the evolutionary mutational patterns these instabilities represent.

The statistical framework formulated above could be useful for evaluating the probability of an artificial mixture of two profiles according to the mtDNA phylogeny versus real instability. The potential of the method can be illustrated by way of a real example. In a study of mtDNA instability in prostate cancer patients, Chen et al. [29] reported the profile of their patient case #1 to have the following variants: A16182C, A16183C, T16189C, C16232A, T16249C, G16274A, T16304C, and T16311C, where all positions were heteroplasmic (that is, a mixture/heteroplasmic-like pattern: 16182X, 16183X, 16189Y, 16232X, 16249Y, 16274R, 16304Y, and 16311Y).The statistical evaluation of this finding can proceed in the conventional way. First, the competing hypotheses are stated as:

- *H_1_: The data is from the patient with some mutations and heteroplasmies added by the tumor.*
- *H_2_: The data is a result of contamination added to the profile of the patient.*

First, note that the contamination hypothesis can be directly dismissed if there are no combinations of haplotypes consistent with the data; thus no calculations are required. Assume, therefore, that *H_2_* cannot be directly rejected. The likelihood of 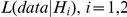 needs to be calculated and we first state the general expressions and subsequently show how this applies to the specific example. Assume there are *S phylogenetic* sites and that the probability of a mutation occurring in site *s* is 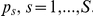 If assumptions can be made on the likelihood of sites for mutations corresponding to hypothesis *H_1_*, then these can be formulated in terms of 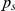. For the applications we are aware of, there are no such assumptions. Rather, mutations occur independently and uniformly. On the condition of there being *x different* mutations, the likelihood is:
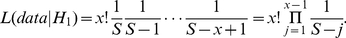
(5)Next, consider the likelihood assuming *H_2_* to be true. In order to make assumptions favorable to hypothesis *H_2_*, only a two-person mixture is considered since including the possibility of contaminations involving more individuals would increase the likelihood. Generally, the likelihood in this case is the sum of the probabilities consistent with the mixture. In this case, there is only one possible contaminating haplotype. Assuming the probability for the contaminating haplotype is 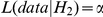, the likelihood ratio in favor of hypothesis *H_2_* becomes:
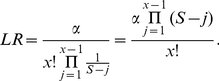
(6)For this specific example, 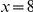 and there are 341 sites. The number of phylogenetic sites, S, is smaller than 341. A conservative calculation is obtained by assigning a lower limit to S, and we use S = 100. In this case, the contaminating haplotype corresponds to rCRS and all conceivable values for 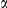 leads to extremely large LR-s. For instance, even a value as low as 0.01 gives an 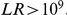 To indicate the strength of the evidence in favor of the contamination hypothesis in this case, even setting 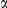 to the lowest known frequency for a haplotype would lead to an extremely large LR value.

## Discussion

We have formulated a formal statistical framework to model mtDNA mixtures that can be applied to real casework cases with the double purpose of (i) unraveling the number of contributors to a mixture and (ii) evaluating the probability of the evidence given the mtDNA profiles of the contributors versus the mtDNA profiles of other individuals different to the contributors.

Several aspects of the models can be refined and further developed as more data and experience become available. For instance, the regression model for the quantitative data requires the error terms to be independent and identically distributed with a truncated normal distribution. These assumptions are particularly important for p-values and confidence intervals to be reliable, whereas estimates of regression coefficients (corresponding to the contributed fractions) are likely to be reasonably valid without these assumptions. Furthermore, the simulations indicate that the results work reasonably well, also when the assumptions are somewhat violated, as in Figure 3. With more data available it will be possible to improve the model for the error distribution (for instance by transforming the data) and also account for the dependence between sites.

Technical issues regarding mtDNA typing were not discussed here but we are aware of the many complications that could complicate the statistical interpretation of real cases. For instance, background noise in sequencing electropherograms could be a hamper mtDNA mixture interpretation. Also, it is not possible to distinguish between two aggressors belonging to the sample matrilineage, given that they share the same mtDNA profile.

Another drawback regarding the quantitative approach is that the Sanger sequencing procedure is not a pure quantitative method. PCR based approaches could distort the relative proportion of each contributor along the electropherogram by preferentially amplifying certain allele variants. If the electropherograms are of good quality, by averaging the proportions of the different variants that participate in the mixture one could estimate the relative proportion of two contributors to the electropherogram (as for instance could be the case in the example illustrated in Figure 4). By way of replicating the PCR amplification and the sequencing procedure, it might be possible to improve the information pertaining to the relative proportion of the different contributors to the mixture. Alternatively, the use of new generation sequencing or methods that allow the sequencing of single strands of DNA would allow a more exact determination of the donor contributions. In such cases, the quantitative statistical approach could be applied without the need for further modifications. The study by Holland et al. [12] is for instance a paradigmatic example. Next generation sequencing needs however proper forensic validation before it can be safely used in forensic casework [30].

It would be useful to provide guidelines on the minimum number of positions that should be queried to reach the level of evidential security needed in court for a conviction. Similarly, it would be helpful to provide recommendations on the minimal number and the positions of mtDNA polymorphisms to be investigated. Simulations can be performed to deal with this problem as mentioned in the section ‘Probability of an informative mixture’ for similar problems. Unfortunately, it is not possible to provide general recommendations on the number of sites needed. The main problem is that the solution would depend on a large number of parameters which are specific for the problem at hand. Rather tailored simulations would have to be done for the specific case based on the database applicable for this problem. Some conclusions and recommendations can be derived from the present study:

- Since mtDNA is inherited as a haplotype block, not all combinations of variants in the mixture are possible (as opposed to the case for unlinked autosomal markers), and therefore phylogenetic inferences and phylogenetic compatibility (the deconvoluted haplotypes should have phylogenetic sense) are also mandatory in order to reduce the universe of haplotypes that could have contributed to the mixture.
- All mixture profiles should be interpreted in light of a known mtDNA phylogeny. If the mixture makes no sense in an evolutionary context, one should suspect the presence of artifacts (for instance due to background noise).
- Using phylogenetics, one could infer the number of contributors in the mixture; however, this is not always possible and depends on the profiles contributing to the mixture.
- As a cautionary note, it is important to highlight the fact that the complications that might arise in mixture cases involving poor DNA samples may well be imponderable; therefore, not all cases can be resolved by way of mtDNA analysis.
- For a generic example we have shown that apparent tumor instabilities are more likely explained in the context of sample mix-up or contamination, which can easily arise in the course of sample preparation or analyses [5].

**Figure 5:**
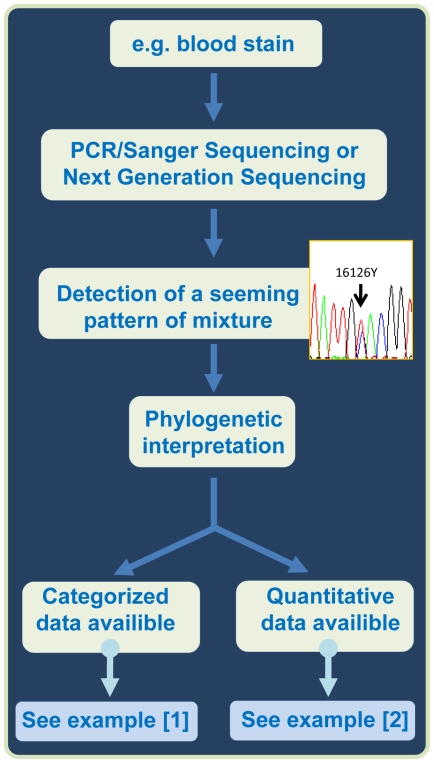
Flowchart of the procedures dealt with in the present study.

Figure 5 shows the flowchart of the procedures dealt with in the present article.

Given that mtDNA analysis is the ultimate choice in mixture cases where autosomal markers have failed, more attention should be given to this maker in complex cases. Herewe have developed a statistical framework and software (freely available from the authors: http://repository.umb.no/R/mtDNA/) that will allow the resolution of cases that otherwise would remain unresolved.
